# Spectroscopic and Morphological Examination of Co_0.9_R_0.1_MoO_4_ (R = Ho, Yb, Gd) Obtained by Glycine Nitrate Procedure

**DOI:** 10.3390/ma18020397

**Published:** 2025-01-16

**Authors:** Milena Rosić, Maja Milošević, Maria Čebela, Vladimir Dodevski, Vesna Lojpur, Uroš Čakar, Srecko Stopic

**Affiliations:** 1 “Vinča” Institute of Nuclear Sciences, National Institute of the Republic of Serbia, University of Belgrade, Mike Petrovića Alasa 12-14, 11351 Belgrade, Serbia; mcebela@vin.bg.ac.rs (M.Č.); vladimir@vin.bg.ac.rs (V.D.); lojpur@vin.bg.ac.rs (V.L.); 2 Faculty of Mining and Geology, University of Belgrade, Đušina 7, 11000 Belgrade, Serbia; maja.milosevic@rgf.bg.ac.rs; 3 Department of Bromatology, Faculty of Pharmacy, University of Belgrade, 11000 Belgrade, Serbia; uros.cakar@pharmacy.bg.ac.rs; 4 IME Process Metallurgy and Metal Recycling, RWTH Aachen University, Intzestrasse 3, 520056 Aachen, Germany; sstopic@ime-aachen.de

**Keywords:** X-ray diffraction, electron microscopy, nanostructured materials, thermochromism

## Abstract

The glycine nitrate procedure (GNP) is a method that proved to be the easiest and most effective method for controlling the composition and morphology during the synthesis of Co_0.9_R_0.1_MoO_4_ (R = Ho, Yb, Gd). This method of the combustion process achieves control of stoichiometry, homogeneity, and purity. Metal nitrates and glycine were mixed in the appropriate stoichiometric ratios to produce Co_0.9_R_0.1_MoO_4_ (R = Ho, Yb, Gd). The samples obtained by the mentioned method were further subjected to different characterization methods such as differential thermal analyses (DTA), X-ray diffraction (XRD), Fourier transform infrared spectrum (FTIR), spectroscopy, field emission scanning electron microscopy (FESEM), and nitrogen adsorption method. A high level of anisotropy of the shape and size of particles in the form of agglomerates was found. Also, there are noticeable differences in the microstructure and plate crystals. The color of the synthesized sample changes from darker to lighter shades after thermal treatments. There are pronounced changes in the dominant wavelength (nm) and color purity between the initial sample and the sample after heating (1100 °C) due to the concentration of Co.

## 1. Introduction

Molybdenia-based ceramic materials have emerged as a significant subject of interest in fundamental research and practical applications. Their unique properties, such as high thermal stability, excellent electrical conductivity, and remarkable mechanical strength, contribute to their appeal [1,2,3,4,5,6,7,8]. These materials are being explored for a wide range of applications, including photoluminescence, which can produce light in response to excitation, and magnetism, which exhibits unique magnetic properties useful in various technologies [1,2,3,4,9,10,11]. Additionally, their roles in catalysis make them valuable for accelerating chemical reactions in industrial processes [5,6,7]. Furthermore, their potential applications in batteries highlight their ability to enhance energy storage and conversion systems, while their conductivity supports advancements in electrical applications [9]. Molybdenum-based ceramics demonstrate significant versatility and innovative potential, making them a critical focus in materials science. Compounds like CoMoO_4_ and other metal molybdates have been extensively studied for their natural abundance, low cost, and non-toxicity [12,13,14]. In addition to CoMoO_4_ showing improved electrochemical properties applied for the manufacture of supercapacitor electrodes, these molybdates found applications in industry, chemistry, electrochemistry, health, energy [15,16,17,18,19,20], and photocatalysts for wastewater treatment and are used in the manufacture of colorants for a larger area of materials (concretes, plasters, paints, soaps, cosmetics, ceramics, glasses…) [21].

Molybdate-based materials have been recognized for their exceptional qualities as host matrices for rare earth ions, demonstrating commendable thermal and chemical stability [22]. Replacing M^2+^/M^3+^ (metal cations) in host materials with rare earth elements has the potential to enhance device stability. Rare earth elements in their trivalent states demonstrate impressive stability over a broad temperature range, providing significant thermal and chemical resilience. By strategically varying the concentration of dopants, we can effectively engineer defects within the crystal structure. This can be accomplished through ion substitution, charge compensation, and introducing oxygen vacancies [22,23,24]. These innovations can potentially enhance the electrochemical properties of the materials, paving the way for more effective applications. For instance, holmium, recognized for having the highest magnetic moment among the lanthanides, and gadolinium, which offers unique metallurgical properties and resistance to high temperatures and oxidation, are noteworthy candidates. Additionally, ytterbium stands out due to its paramagnetic characteristics at temperatures above 1K, making it an interesting option. Exploring these elements may lead to advancements in material performance and application.

The study and development of thermochromism and thermochromic materials hold immense potential, driving innovation across diverse fields such as energy-efficient building design, textile industries, thermal storage, antique preservation, and sensory technology [25]. Thermochromic materials exhibit a reversible color change in response to temperature variations, although there are instances where the changes may be irreversible. This change occurs at a specific temperature, referred to as the thermochromic transition. It can be defined by an observable color change induced by a shift in molecular conformations due to a temperature change, which can be thoughtfully modified by incorporating doping agents into the material, unlocking endless possibilities for creativity and innovation. Various materials exhibit different responses to specific stimuli; nonetheless, the mechanism underlying these color changes generally involves a reversible transfer of electrons or ions. This process requires energy from an external source to overcome potential barriers effectively [26,27]. This study explores the thermochromic potential of cobalt molybdate by incorporating Ho, Yb, or Gd as a dopant. While various cobalt molybdates have been investigated, there appears to be a lack of literature regarding nanoscale CoMoO_4_ doped with trace amounts of rare earth elements such as the investigated ones presented in this manuscript. Previous research has involved rare earth doping in systems of Cd and Ca [22,28,29]; however, these studies do not pertain to the novel Co_0.9_R_0.1_MoO_4_ (R = Ho, Yb, Gd) nanopowders. This work emphasizes the novelty of using the glycine nitrate process (GNP) [30] to synthesize nanoparticles to advance research in this domain. The primary objective is to synthesize nanostructured solid solutions of the specified composition and analyze their spectroscopic and morphological properties using various experimental techniques, thereby providing new insights into the nature and characteristics of these materials.

## 2. Materials and Methods

### 2.1. Powder Preparation

In order to have better control over the stoichiometry, structure, and phase purity of metal oxides, the concept of avoiding the brute force method is still resorted to during the synthesis of new materials. When applying the glycine nitrate method, it is important to prioritize the molar ratio of glycine to nitrate ions in the mixture. A higher reaction temperature (exothermicity) will undoubtedly lead to larger crystallite sizes in the final material, which means that the starting reactants will react more effectively. Focusing on these factors is essential for achieving optimal results. Starting chemicals used for the GNP (glycine nitrate procedure) synthesis of powders were aminoacetic acid–glycine NH_2_CH_2_COOH (Fischer Scientific, Waltham, MA, USA), Co-nitrate Co(NO_3_)_2_ · 6H_2_O (Sigma-Aldrich, A Part of Merck, KGaA, Darmstadt, Germany), Ho-nitrate Ho(NO_3_)_3_ · 5H_2_O (Sigma-Aldrich, A Part of Merck, KGaA, Darmstadt, Germany), Yb-nitrate Yb(NO_3_)_3_ · 5H_2_O (Sigma-Aldrich, A Part of Merck, KGaA, Darmstadt, Germany), Gd-nitrate Gd(NO_3_)_3_ · 6H_2_O (Sigma-Aldrich, A Part of Merck, KGaA, Darmstadt, Germany) and ammonium molybdate (NH_4_)_6_Mo_7_O_24_ · 4H_2_O (Sigma-Aldrich, A Part of Merck, KGaA, Darmstadt, Germany), all >99.9% purity. The principle of propellant chemistry [31] was used to calculate the composition of the reacting mixtures. According to the established principles of propellant chemistry, it is essential to recognize that in stoichiometric redox reactions between a fuel and an oxidizer, the ratio of the net oxidizing valences of the metal nitrate to the net reducing valences of the fuel must be uniquely defined. This insight is crucial for advancing our understanding and applications in this specialized field. By convention, the valencies of carbon, nitrogen, oxygen, hydrogen, cobalt, holmium, ytterbium, gadolinium, and molybdenum are set as 4+, 0, 2–, 1+, 2+, 3+, 3+, 3+ and 6+, respectively. Using the valencies of these individual elements, the reducing valence of glycine C_2_H_5_NO_2_ = 2(+4) + 5(+1) + 0 + 2(−2) = 9; it works out to be 9+, while the net oxidizing valencies of Co(NO_3_)_2_ and (NH_4_)_6_Mo_7_O_24_ work out to be 10– and 18+, respectively. Ho(NO_3_)_3_, Yb(NO_3_)_3_ and Gd(NO_3_)_3_ work out to be 15–, respectively. We add these values based on Equations (1)–(3) and divide by 9 (the value for glycine). For producing one mole of Co_0.9_Ho_0.1_MoO_4_, Co_0.9_Yb_0.1_MoO_4,_ and Co_0.9_Gd_0.1_MoO_4_, through the stoichiometric reactions (1), (2), and (3), there will be −7.93/9 = 0.88 moles of glycine, which is required. According to the chemical equations, Co_0.9_R_0.1_MoO_4_ (R = Ho, Yb, Gd) powders were prepared:

C_2_H_5_NO_2_ + 0.9Co(NO_3_)_2_ · 6H_2_O + 0.1Ho(NO_3_)_2_ · 5H_2_O + 0.14(NH_4_)_6_Mo_7_O_24_ · 4H_2_O + 2.2O_2_ → Co_0.9_Ho_0.1_MoO_4_+ CH_3_COOH + 3.96NO_2_↑ + 8.69H_2_O (1)

C_2_H_5_NO_2_ + 0.9Co(NO_3_)_2_ · 6H_2_O + 0.1Yb(NO_3_)_2_ · 5H_2_O + 0.14(NH_4_)_6_Mo_7_O_24_ · 4H_2_O + 2.2O_2_ → Co_0.9_Yb_0.1_MoO_4_+ CH_3_COOH + 3.96NO_2_↑ + 8.69H_2_O (2)

C_2_H_5_NO_2_ + 0.9Co(NO_3_)_2_ · 6H_2_O + 0.1Gd(NO_3_)_2_ · 6H_2_O + 0.14(NH_4_)_6_Mo_7_O_24_ · 4H_2_O + 2.2O_2_ → Co_0.9_Gd_0.1_MoO_4_+ CH_3_COOH + 3.96NO_2_↑ + 8.74H_2_O (3)

The samples were obtained based on the calculations according to the principle of propellant chemistry and the reaction model presented by Equations (1)–(3).

According to the previously calculated composition of the final powder, the synthesis was carried out in a stainless-steel reactor in which all reactants dissolved in distilled water were added. When the reactants are dissolved in water, the reaction starts initially in this heterogeneous medium at the interface [32]. The reaction begins spontaneously at 180 °C. The reactants are heated to 500 °C because, at this temperature, gas evolution ceases, indicating the reaction’s completion. During the reaction, the loss of powders was small, and the amount of powders obtained was very close to the calculated value. The obtained powders were further calcined for 15 min at temperatures of 450 and 1100 °C.

### 2.2. Sample Characterization

Thermal properties have been investigated by a differential thermal analyses furnace, A.D.A.M.E.L., equipped with Pt-PtRh thermocouples and a digital data acquisition computer system. The *t* and Δ*t* voltages were collected over a 16-bit USB-2523 AD converter with a 1 Hz sampling rate. The heating rate was 8 °C/min in a temperature range from room temperature to 1100 °C. The reference material used was α-Al_2_O_3,_ according to which the baseline was corrected.

X-ray diffraction (XRD) patterns of the samples were recorded at room temperature by X-ray diffractometer Rigaku Ultima IV equipped with CuKα_1,2_ radiations using a generator voltage (40.0 kV) and a generator current (40.0 mA). The range of 10–80° 2*θ* was used for all powders in a continuous scan mode with a scanning step size of 0.02° and at a scan rate of 10°/min, using the D/TeX Ultra-high-speed detector. A glass sample carrier for sample preparation was used. The PDXL2 (Ver. 2.8.4.0) software was used to evaluate the phase composition and identification [33]. All obtained powders were identified using the ICDD database [34] and the ICSD database [35]. Structural analysis was carried out by using the program PowderCell 2.4 [36,37]. The TCH pseudo-Voigt profile function gave the best fit to the experimental data.

Fourier transform infrared spectrum (FTIR) spectra of the prepared and calcinated samples were performed with FTIR BOMEM (Hartmann and Braun). KBr pellets were made for all the powders. The spectra were recorded in the region 4000–400 cm^−1^ with a resolution of 2 cm^−1^.

The dominant color wavelength (nm) of the dry-pressed, raw powder sample before and after firing at 1100 °C was measured using a CCS200 spectrometer (Thorlabs, Newton, NJ, USA), equipped with diffuse reflectance apparatus, in the spectral range 400–700 nm using BaSO_4_ as a blank. Optical fiber guided light from the lamp to a planoconvex lens, which focused the emitted light to a spot size of around 2 mm in diameter on the sample’s surface. Investigations were made at room temperature, and the mean value of three measurements was represented as the final result. Color investigations were carried out according to the Commission Internationale de l’Eclairage (1932) [38]. Using the freeware Radiant Imaging Color Calculator program, the chromaticity coordinates were expressed on a 2D chromaticity CIE color diagram [39].

The morphology of samples was estimated from Field emission scanning electron microscopy (FESEM) micrographs (FEI Scios 2, operating voltage of 30 kV). The chemical composition of samples was examined by energy-dispersive X-ray spectroscopy (EDS).

The specific surface and pore size distribution were studied by the low-temperature nitrogen (N_2_) adsorption–desorption isotherm tests according to the Brunauer Emmett Teller (BET) method using a surfer gas adsorption porosimeter (Thermo Fisher Scientific, Waltham, MA, USA). The specific surface area, *S*_BET_, pore size distribution, d*V*(*r*), mesopore including external surface area, *S*_meso_, and specific micropore volume, *V*_mic_, for the sample were calculated from the full adsorption/desorption isotherm. Pore size distribution d*V*(*r*) was calculated from the desorption isotherm branch by applying the Barrett, Joyner, and Halenda (BJH) method [40], while a mesopore surface and micropore volume were estimated using the *t*-plot method [41].

## 3. Results and Discussion

### 3.1. Differential Thermal Analyses (DTA)

**Figure 1:**
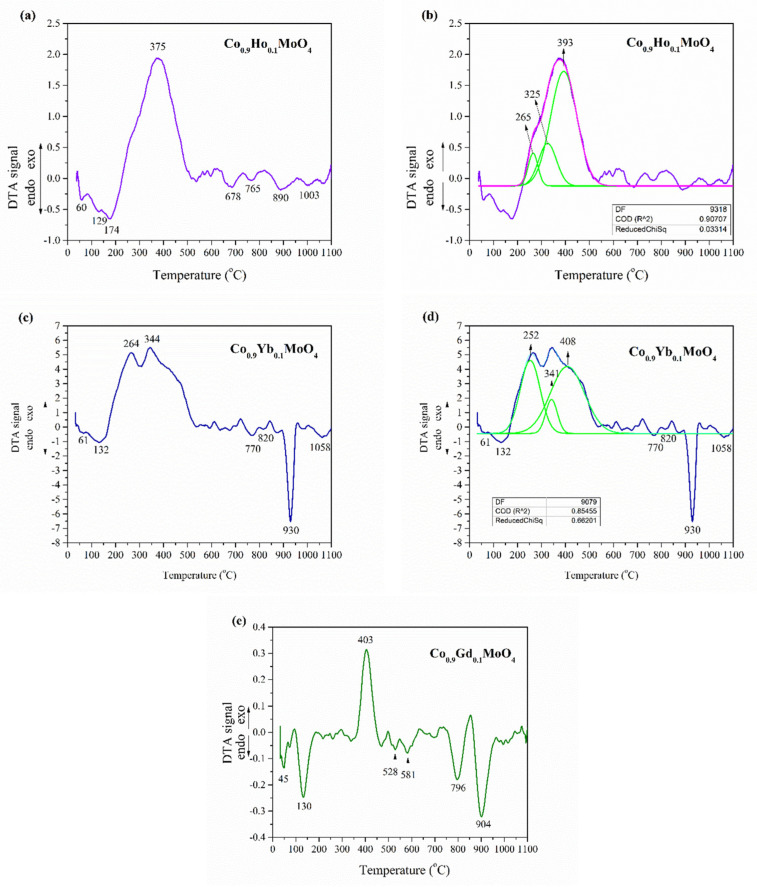
DTA curve for the synthesized powder of Co_0.9_R_0.1_MoO_4_ (R = Ho, Yb, Gd): (**a**,**b**) Co_0.9_Ho_0.1_MoO_4_; (**c**,**d**) Co_0.9_Yb_0.1_MoO_4_; (**e**) Co_0.9_Gd_0.1_MoO_4_; (**b**,**d**) Application Gaussian peak functions from OriginPro 8.5 (green lines).

Investigated samples have shown several reactions occurring at different temperature ranges: between 60 and 170 °C, 250 and 450 °C, and above 650 °C, with notable bands around 760 °C, 890 °C, and between 900 and 1000 °C (Figure 1). To determine the positions of these reactions, Gaussian peak functions from OriginPro 8.5 were applied. The results showed the band intensities, particularly in samples doped with Ho (Figure 1b). and Yb (Figure 1d), revealed a strongly exothermic reaction that created a broad peak between 250 and 450 °C, suggesting the possibility of additional reactions. The sample doped with Gd was also investigated using the same peak functions; however, due to the narrowness of the band, it did not display any additional peaks. The first transformation begins with endothermic reactions indicating the removal of crystallization water from cobalt oxide, occurring between 45 and 170 °C. These reversible reactions signify water loss from the powder mixture [30,40,42]. The first decomposition phase of Co_0.9_Ho_0.1_MoO_4_ starts with an endothermic reaction at 129 °C, followed by another endothermic reaction at 174 °C, and then a rapid exothermic reaction peaking at 375 °C (Figure 1a,b). This indicates the start of the second phase, where NO_3_^−^ and glycine begin to evolve simultaneously [30]. For the second sample, Co_0.9_Yb_0.1_MoO_4_, the first phase transformation starts with endothermic water loss reactions at 61 °C, followed by another endothermic reaction at 132 °C, which is succeeded by a rapid exothermic reaction at 264 °C (Figure 1c,d). The third sample, Co_0.9_Gd_0.1_MoO_4_, exhibited several endothermic reactions similar to the previously discussed ones, with endothermic reactions at 45 °C and 130 °C, indicating the loss of water molecules (Figure 1e). It was observed that the sample doped with Ho exhibited greater water loss, as noted in higher band intensity, compared to those doped with Gd and Yb, which showed similar levels of loss when compared to each other. Like the previous, the samples doped with Ho and Yb exhibited a rapid exothermic reaction at 405 °C, marking the initiation of the second stage with the simultaneous release of NO^3−^ and glycine. The exothermic band observed between 260 and 410 °C indicates the formation of a stable compound due to increased heat and corresponds to the complete breakdown of the initial mixture, resulting in a strong combustion reaction and total loss of coordination water [30,42,43]. There is no visible endothermic band at the 250 °C mark, which has been reported to coincide with the complete decomposition of glycine [44]. Similar reactions were reported by Lai et al. [45] during the low-temperature synthesis of LiNi_1-x_Co_x_VO_4_. Furthermore, the broad peak observed between 325 and 450 °C in the sample doped with Ho suggests not only the crystallization of the *β*-CoMoO4 phase but also the oxidation of residual carbon from glycine [30,43,46,47]. The endothermic bands between 670 and 890 °C could indicate a possible transition to *α*-CoMoO4. The endothermic peak at 770 °C, along with a shoulder at 820 °C, indicates the melting of MoO_3_, which decomposes at around 790 °C [48,49]. The third transformation stage exhibits a strong endothermic reaction between 900 and 1000 °C, possibly suggesting a transition from the *β* phase to the *α* phase [30,43]. The sample doped with Ho shows a significantly lower intensity of the band corresponding to this third transformation when compared to the other samples.

### 3.2. XRD Analysis

**Figure 2:**
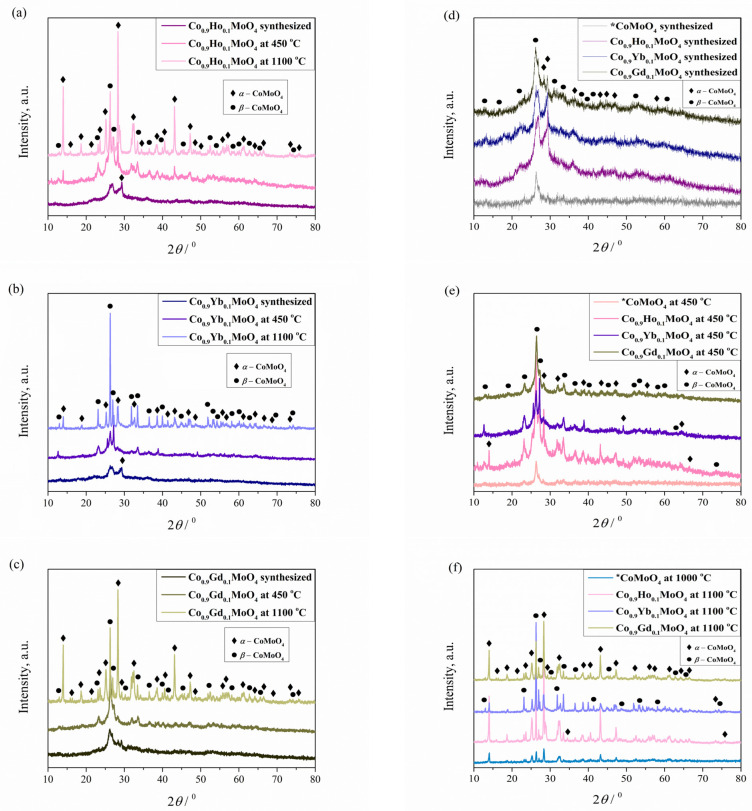
XRD pattern of Co_0.9_R_0.1_MoO_4_ (R = Ho, Yb, Gd) powder sample after combustion reaction: (**a**) Co_0.9_Ho_0.1_MoO_4_ (**b**) Co_0.9_Yb_0.1_MoO_4_ (**c**) Co_0.9_Gd_0.1_MoO_4_; * undoped CoMoO_4_ [30]; (**d**) comparison of undoped synthesized CoMoO_4_ with doped synthesized Co_0.9_R_0.1_MoO_4_ (R = Ho, Yb, Gd) samples; (**e**) comparison of undoped CoMoO_4_ at 450 °C with doped Co_0.9_R_0.1_MoO_4_ (R = Ho, Yb, Gd) samples at 450 °C; (**f**) comparison of undoped CoMoO_4_ at 1000 °C with doped Co_0.9_R_0.1_MoO_4_ (R = Ho, Yb, Gd) samples at 1100 °C.

The XRD patterns of the Co_0.9_Ho_0.1_MoO_4_, Co_0.9_Yb_0.1_MoO_4,_ and Co_0.9_Gd_0.1_MoO_4_ powders were obtained after combustion reaction (GNP) and calcination at 450 and 1100 °C are shown in Figure 2a–c. The percentage of phases and unit cell parameters of the newly formed compound were determined based on XRD data (Table 1). For all three systems present, it was established that two-phase samples were obtained, representing two polymorphic modifications that simultaneously crystallized in the same monoclinic space group, C*2*/*m*. Phase transitions and polymorphic modifications are characteristic of CoMoO_4_ powders [50], which were not absent in our case, despite the doping of CoMoO_4_ with a small amount of holmium, ytterbium, and gadolinium. Based on previously conducted research related to the same applied synthesis for undoped CoMoO_4_ [30], it was expected that only one polymorphic *β* modification would be formed after the synthesis. However, due to doping with Ho, Yb, and Gd during the combustion reaction, we achieved the presence of both modifications at all tested temperatures in unequal proportions. The XRD patterns for the synthesized samples (Figure 2d) and those related to the samples calcined at 450 °C (Figure 2e), the presence of two peaks at 26.1–26.4 and 29.2 degrees was observed, thus identifying two resulting phases, which represent two polymorphic modifications: *α*-CoMoO_4_ (base-centered monoclinic space group C*2/m*, no. 12, ICSD #23808, corresponds to 28.5 degrees, and Miller indices [220]) and *β*-CoMoO_4_ (monoclinic space group C*2/m*, no. 12, ICSD #78328, corresponds to 25.7 degrees, and Miller indices [220]) [35]. By identifying the samples heat-treated at 1100 °C (Figure 2f), we assumed that the *α*-isomorph was the only phase since the *α*-phase was reported to be stable above 1000 °C [24,49,51], but the *β*-isomorph was also present. The diffraction peaks we studied (Figure 2) agree well with the values given in the ICSD cards, with the difference that there was a minor shift of the peaks to one of the sides and an increase in their intensities. According to X-ray measurements, the XRD patterns of all powders appeared similar to each other for the same calcination temperatures despite the doping with different rare earth elements. The only noticeable difference in the Yb-doped samples relates to the proportion of phases. Based on peaks with pronounced intensities, the β polymorph is the dominant phase in the Yb-doped samples (Table 1).

The diffraction peaks seen in Figure 2 are broad and of low intensity for the samples synthesized and calcined at a temperature of 450 °C, which indicates the nanocrystalline nature of the powders. However, the sample calcined at 1100 °C is also visually different, with a significantly higher degree of crystallinity. The XRD analysis of the samples confirmed that Co_0.9_R_0.1_MoO_4_ (R = Ho, Yb, Gd) exhibits thermochromic behavior and that the temperature factor plays an important role in forming *α*, *β*, or both polymorphs.

We compared the parameters of the unit cells of the samples, synthesized and calcined at 450 or 1100 °C with the composition of Co_0.9_R_0.1_MoO_4_ (R = Ho, Yb, Gd) (Table 1), and before the published date for undoped CoMoO_4_ [30]. We did not find any regularity in the changes in unit cell parameters. The presence of both polymorphs at temperatures where they should be the only phases is evident. By substituting part of Co^2+^ (r_ion_(Co^2+^) = 0.745 Å) with Ho^3+^ (r_ion_(Ho^3+^) = 1.041 Å), or with Yb^3+^ (r_ion_(Yb^3+^) = 1.008 Å), or with Gd^3+^ (r_ion_(Gd^3+^) = 1.078 Å), there was a significant impact on the temperature of the phase transition [24]. The reason for this is the presence of Ho^3+^, Yb^3+^, or Gd^3+^ in the system, which creates Mo–O covalent bonds but not enough to stabilize Mo^6+^ in a tetrahedral environment with stabilization of the *β* form, which is why the *β*→*α* transition occurs at higher temperatures [24].

### 3.3. FTIR

**Figure 3:**
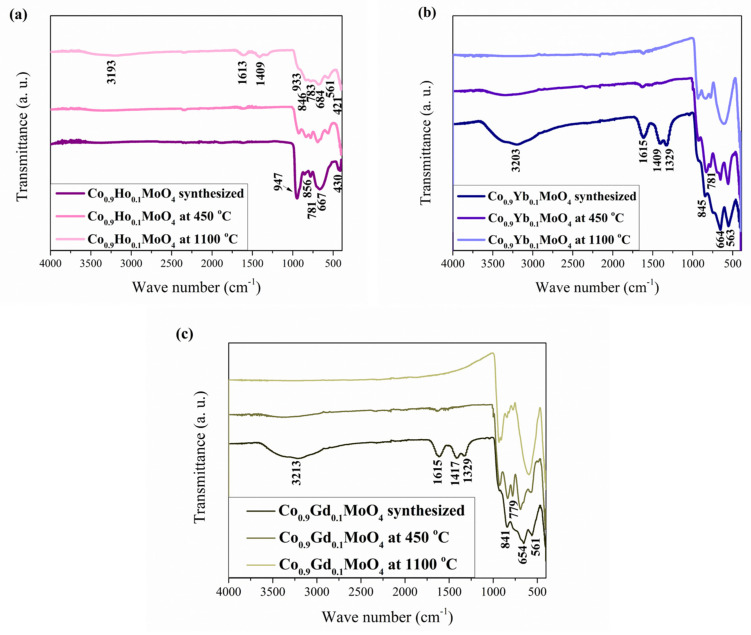
The FTIR spectra of the as-prepared Co_0.9_R_0.1_MoO_4_ (R = Ho, Yb, Gd) samples: (**a**) Co_0.9_Ho_0.1_MoO_4_ (**b**) Co_0.9_Yb_0.1_MoO_4_ (**c**) Co_0.9_Gd_0.1_MoO_4_.

The prepared Co_0.9_Ho_0.1_MoO_4_, Co_0.9_Yb_0.1_MoO_4,_ and Co_0.9_Gd_0.1_MoO_4_ samples were characterized by FTIR spectroscopy to investigate the nature of the bonding and understand the structural differences between them, as shown in Figure 3a–c. In the first three spectra (Figure 3a), which correspond to Co_0.9_Ho_0.1_MoO_4_ samples, a weak band at ~2350 cm^−1^ attributed to physisorbed CO_2_ can be observed [52]. The two bands present at 1610 and 1415 cm^−1^ represent the stretching and bending vibrations of the O–H and H–O–H bonds due to the absorbed water molecules from atmospheric moisture [53] and the nitrate band and binding of the primary amine (i.e., H_2_N–CH_2_-) [54]. Otherwise, during the combustion reaction, glycine has a double role; it acts as a fuel and also as a complexant. The interaction of metal cations completely complexed with glycine is confirmed by the band at 1415 cm^−1^ [54]. The spectrum shows three main bands at 950, 665, and 560 cm^−1^. The two vibrational bands at 950 cm^−1^ and 665 cm^−1^ are attributed to the activation of the *ν*_1_ vibration of the distorted MoO_4_ tetrahedral units present in CoMoO_4_ [30], while the band at 560 cm^−1^ is due to the Ho-O species [55]. Other vibrational bands at 840 and 779 cm^−1^ are ascribed to the stretching vibration of Mo–O–Mo. The superposition of ν_4_ and *ν_5_* of MoO and *ν_3_* of CoO_6_ building groups of CoMoO_4_ was attributed to the band at 418 cm^−1^ [30,56].

The second three spectra (Figure 3b), which correspond to the Co_0.9_Yb_0.1_MoO_4_ samples, and the third three spectra (Figure 3c), which correspond to the Co_0.9_Gd_0.1_MoO_4_ samples, are more similar to each other, and bands that are not present in the Co_0.9_Ho_0.1_MoO_4_ samples can be observed in them. Namely, weak absorption bands extend in the ~3200 cm^−1^ range, which is attributed to the H–O–H groups of water molecules formed by humidity [53,57]. The two bands present at 1610 and 1415 cm^−1^ represent the stretching and bending vibrations of the O–H and H–O–H bonds [52], and the nitrate band and binding of the primary amine (i.e., H_2_N–CH_2_–) [54] are more pronounced in those samples. For spectrums from Co_0.9_Yb_0.1_MoO_4_ samples, the characteristic band around 545 cm^−1^ confirms the stretching vibrations of Yb ions [58]. The bands around 542 and 440 cm^−1^ assigned to the Gd-O vibration [59] are characteristic of spectrums from Co_0.9_Gd_0.1_MoO_4_ samples.

### 3.4. FESEM Analysis

**Figure 4:**
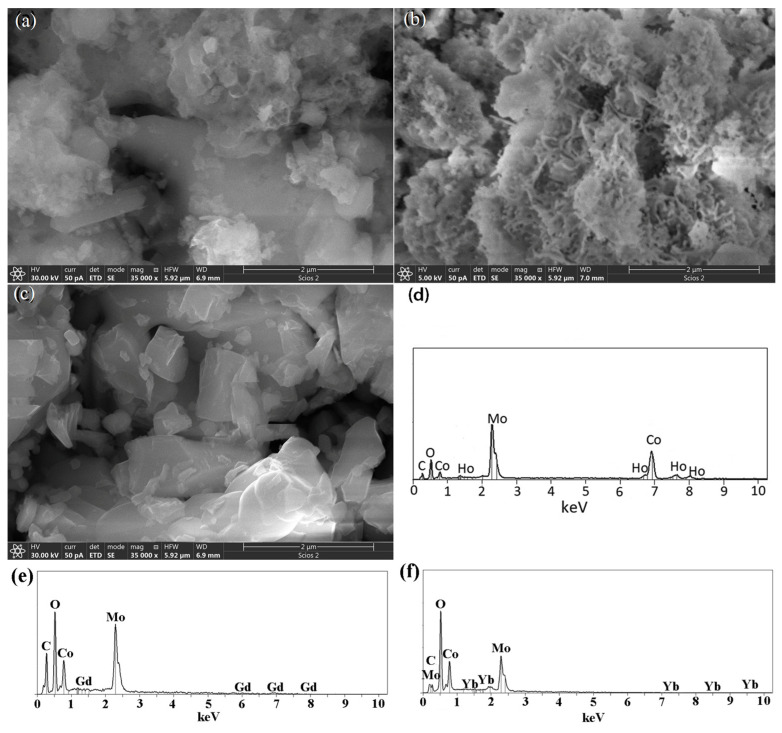
FE-SEM micrographs of Co_0.9_Ho_0.1_MoO_4_ as-prepared sample (**a**); (**b**) at 450 °C; (**c**) at 1100 °C; (**d**) one representative EDS spectrum of Co_0.9_Ho_0.1_MoO_4_; (**e**) one representative EDS spectrum of Co_0.9_Gd_0.1_MoO_4_; (**f**) one representative EDS spectrum of Co_0.9_Yb_0.1_MoO_4_.

The information on the morphology and chemical analysis of the samples was provided by FE-SEM together with EDS analysis and presented in Figure 4. One can see the micrographs of as-prepared as well as calcined samples at 450 and 1100 °C (Figure 4a–c), recorded using the same experimental conditions. A high level of shape anisotropy and particle size in the form of agglomerates is observed. Also, microstructure differences are clearly noticeable in all three samples. The as-prepared samples tend to form irregular agglomeration with a grape-like structure (Figure 4a). Irregular agglomerations consist of polyhedral grains and plate-like crystals of various sizes, and between them are interstices, cavities, and voids. On the other hand, the sample calcined at 450 °C shows leaf-like nanosheets (Figure 4b) forming plate-acicular agglomerated grains with too many occasionally connected pores. The pores are most likely the result of a large amount/volume of gases that evolved during this synthesis method [60]. A sample calcined at 1100 °C possesses large, irregular, polyhedral grains (Figure 4c) with smooth and non-porous surfaces. Pores and cavities are almost absent in these samples, which will be evident and confirmed during further characterization methods and will be presented in this article. Because the morphology of the other two sets of samples was quite identical, only EDS spectra were presented to demonstrate the inclusion of constituent elements.

In order to confirm the presence of wanted ions, EDS spectra were recorded in the energy range of 0–10 eV for all samples, and only one representative EDS spectrum was presented in Figure 4d,e for each Co_0.9_R_0.1_MoO_4_ (R = Ho, Yb, and Gd). The spectra confirmed that all Co, Mo, O, and R elements are present in the structure (Table 2).

### 3.5. Spectroscopy

The color of the synthesized samples of Co_0.9_Ho_0.1_MoO_4_, Co_0.9_Yb_0.1_MoO_4_, and Co_0.9_Gd_0.1_MoO_4_ was visually observed as a dark blue, dark purple, and black tint, respectively. After spectrophotometric measurement of the color, it was noted that the dominant wavelengths were 427, 456, and 469 nm for samples with Ho, Yb, and Gd, respectively. Color saturation is most pronounced after doping with Ho, reaching 99.8%; Yb doping results in around 65% saturation, while the least saturated sample, 13%, is the one doped with Gd (Table 3).

**Figure 5:**
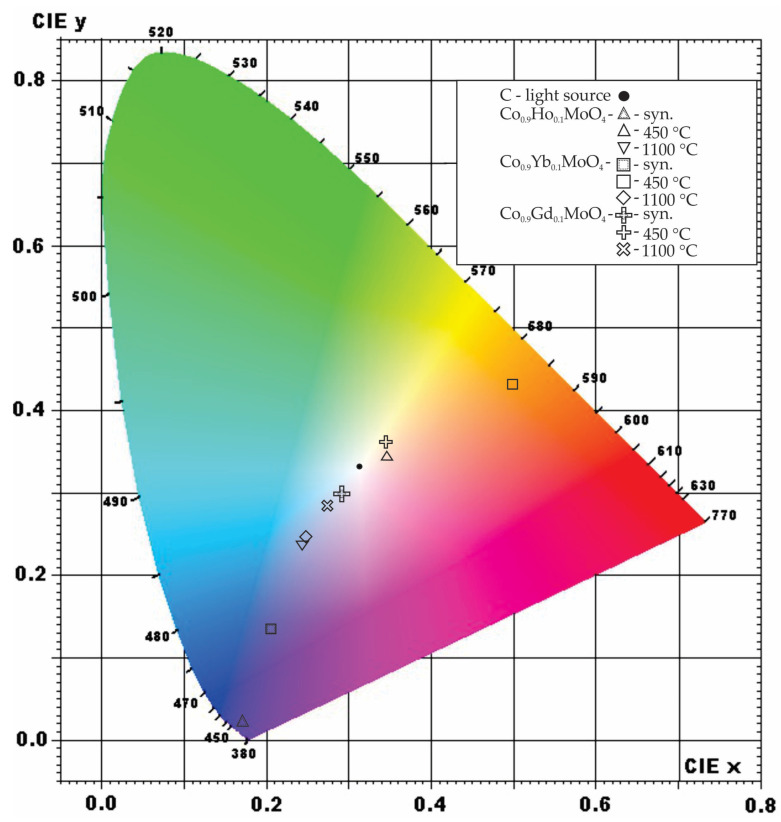
Chromatic diagram of Co_0.9_R_0.1_MoO_4_ (R = Ho, Yb, Gd) samples.

After subjecting the synthesized sample to heating treatment, there was a complete change in the color and intensity (Table 3). The dark purple/black shade, after heating at 450 °C, shifts to a higher wavelength with lower color purity, 589.02 nm (Dc) and 4.4% (Pc) for the sample doped with Ho. The transition that occurs in this sample corresponds to the transition that occurs after heating. The measured wavelength of 589.02 nm falls between yellow and orange-red light, although in that spectral area, it was noted that purple and bluish/black tints also appear [61]. Wavelengths at around 580 nm correspond to the transmitted color, which is visually observed as violet, blue, or green (Figure 5). The pronounced change in dominant wavelength and the purity of the color between the synthesized sample with Ho and the same sample after the heating at 1100 °C is due to Co concentrations. When aluminum oxide is heated with cobalt compounds or when cobalt salt is precipitated with an alkali, a bright blue precipitate forms from an oxidized black starting compound [62]. The addition to the blue color shift gives molybdenum content that, after heating, leads to the formation of the so-called molybdenum blues. The blue color is due to the presence of colored cations, violet cobalt (II) molybdate, CoMoO_4_ [63,64].

A dominant wavelength of 456 nm was measured for the synthesized sample doped with Yb, which corresponds to the dark blue region of the visible spectrum (380 nm to 750 nm), with a color purity of 65.3% (Figure 5). Similar to the previous sample doped with Ho, this color is due to the concentration of cobalt and molybdenum compounds, which produce dark blue colors [62,63,64]. The color change, occurring after heating, is due to the presence of Yb, causing a dark blue–green color shift after heating, which was too saturated for absorption measurements. Therefore, the dominant wavelength shifted to 585 nm with a color purity of 4.39%, corresponding to the yellow region of the visible spectrum (570 nm to 590 nm). This color represents the transmitted color after absorption of other colors, and it is directly opposite on the diagram from the dark blue spectra (corresponding to ≈485 nm). Ytterbium, which is white, and its salts are colorless in visible light, would produce an emerald green or even orange color at very high temperatures (3000 °C) [65]. A similar shift of color was observed after heating the sample at 1100 °C with the color corresponding to 472 nm (PC = 32.8%) in the blue–purple area with its corresponding color in the yellow–orange region (Figure 5). This suggests that a small amount of Yb would cause a slight shift in its color from dark blue to the purple-blue end of the spectrum after heating.

The color of the synthesized sample doped with Gd closely resembles the previously mentioned samples doped with Ho and Yb. A slight change is observed due to the dominant wavelength, which is 469 nm, corresponding to the dark blue region [62,63,64]. This makes the sample lighter, and its purity color percentage corroborates it at 13.1%. The color change occurs after heating at 450 °C, resulting in a yellow hue in the visible spectrum corresponding to the absorption of its complementary color, dark blue–purple color, and correlates to the reaction of Gd with other elements in the sample. After heating at 1100 °C, the investigated sample yields a color with the dominant wavelength of 475 nm (PC = 30.3%), which is closely similar to the previously investigated samples (Table 3).

### 3.6. BET Analysis

**Figure 6:**
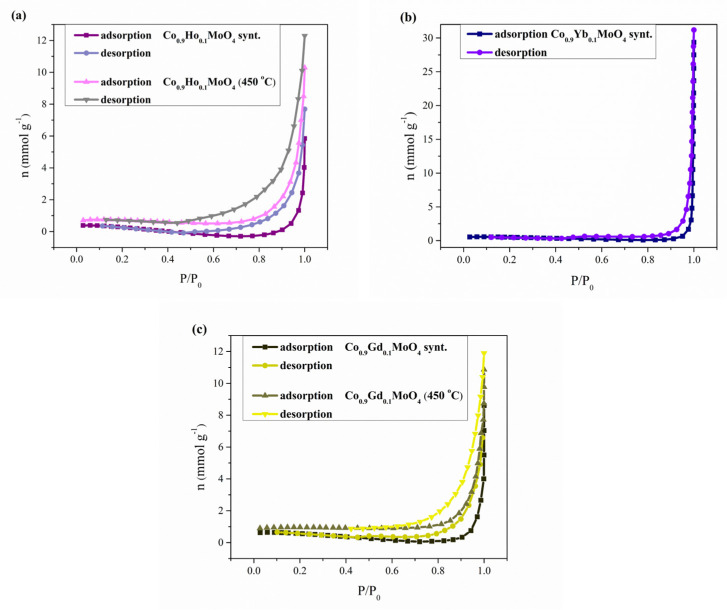
Nitrogen adsorption isotherms, as the amount of N_2_ adsorbed as a function of relative pressure for: (**a**) Co_0.9_Ho_0.1_MoO_4_ samples synthesized and at 450 °C; (**b**) Co_0.9_Yb_0.1_MoO_4_ samples synthesized; (**c**) Co_0.9_Gd_0.1_MoO_4_ samples synthesized and at 450 °C.

The specific surface areas of Co_0.9_R_0.1_MoO_4_ (R = Ho, Yb, Gd) samples, synthesized and calcined at 450 and 1100 °C, were measured by BET (Brunauer–Emmett–Teller) analysis using the nitrogen adsorption analysis data shown in Figure 6. All investigating samples calcined at 1100 °C and Co_0.9_Yb_0.1_MoO_4_ calcined at 450 °C did not show a specific surface area; therefore, in the following text, figure, and table, data related to these measurements are missing. If we return to Figure 4c, where the absence of pores was observed, the result of the BET analysis could be guessed. Figure 6. shows the full adsorption/desorption isotherm of nitrogen adsorbed on the sample’s surface. Nitrogen adsorption isotherms for Co_0.9_Ho_0.1_MoO_4_ and Co_0.9_Gd_0.1_MoO_4_ samples, synthesized and calcined at 450 °C, and only Co_0.9_Yb_0.1_MoO_4_ samples synthesized as the amount of N_2_ adsorbed as a function of relative pressure at −196 °C, are shown in Figure 6.

The obtained isotherms are characterized by pronounced capillary-condensation hysteresis and belong to type IV isotherms attributed to monolayer–multilayer adsorption according to the IUPAC classification [66]. They are corresponding to mesoporous materials. The samples have the shape of the hysteresis loop of type H3. Isotherms revealing type H3 hysteresis do not exhibit any limiting adsorption at high P/P_0_ and are observed with non-rigid aggregates of plate-like particles, giving rise to slit-shaped pores [67]. The observed hysteresis consists of one loop. The hysteresis loop (in the region of relative pressure 0.4 < P/P0 < 1) can be attributed to the classical type H3, which indicates the presence of bottle-shaped mesopores. Table 4 presents the values for the specific surfaces of the examined samples calculated by the BET equation, S_BET_. S_BET_ values for Co_0.9_Ho_0.1_MoO_4_ samples are in the range of 3 to 14 m^2^ g^−1^; for Co_0.9_Gd_0.1_MoO_4_ samples, they are in the range of 11 to 16 m^2^ g^−1^; and for the Co_0.9_Yb_0.1_MoO_4_ sample, 10 m^2^ g^−1^.

Pore size distribution (PSD) of Co_0.9_Ho_0.1_MoO_4_ and Co_0.9_Gd_0.1_MoO_4_ samples synthesized and calcinated at 450 °C is shown in Table 4. The pore radius of the samples varies between 9 and 11 nm, confirming the mesoporous structure of both samples. As indicated by the volume of micropores, all samples have only a small amount of micropores. The median pore radius for all samples is given in Table 4.

## 4. Conclusions

Over the years, thermochromic materials have been intensively studied for their wide-ranging applications in modernization, urbanization, and improving healthcare systems. These materials require investigation through synthesis and processing routes to ensure they are more efficient and durable when used in specific applications [27]. Despite its limitations, this study’s findings support the following conclusions:

The combustion process described here through the glycine nitrate procedure (GNP) is one of the innovative material processing techniques. Co_0.9_R_0.1_MoO_4_ (R = Ho, Yb, Gd) samples were successfully synthesized using this method in a technically simple, fast, and low-cost way.

A comparative analysis was applied for the newly obtained powder characterization using several experimental techniques. The DTA curve for the synthesized Co_0.9_R_0.1_MoO_4_ (R = Ho, Yb, Gd) powders indicates that the doping with holmium, ytterbium, and gadolinium caused a change in the temperature ranges for polymorphic transitions. So, the possibility of the creation of *β*-Co_0.9_R_0.1_MoO_4_ (R = Ho, Yb, Gd) is in the temperature range from 325 to 450 °C, and the formation of α-Co_0.9_R_0.1_MoO_4_ (R = Ho, Yb, Gd) in the form of polymorphic transition *β*→*α* at 1003 °C.

XRD structural analysis of the investigated samples established that Co_0.9_R_0.1_MoO_4_ (R = Ho, Yb, Gd) powders crystallize in the monoclinic space group C2/m, no. 12. Two structural types appear, designated like polymorphs *α* and *β*, whose amount of polymorphs varies with increasing temperature.

The influence of holmium, ytterbium, and gadolinium as dopants led to significant changes in the temperature range of polymorph formation and affected its morphological properties. The tendency of agglomeration and an inhomogeneous microstructure with plate crystals have been retained.

Following the heat treatment of the synthesized sample, we observed a notable change in color and intensity. The dominant wavelength (Dc) exhibited an increase for all samples treated at 450 °C, and interestingly, even the samples subjected to 1100 °C showed a higher dominant wavelength than the synthesized samples. It is important to note that the color purity (Pc) varied depending on the dopant element. Specifically, the synthesized Co_0.9_Ho_0.1_MoO_4_ demonstrated the highest color purity, succeeded by Co_0.9_Yb_0.1_MoO_4_ at 450 °C, while Co_0.9_Gd_0.1_MoO_4_ at 1100 °C exhibited a comparatively lower level of purity.

The mesoporous structure was confirmed in the synthesized and calcined samples at 450 °C; the samples and the surface area are between 3 and 16 m^2^g^−1^, while we could not determine it for the samples calcined at 1100 °C for all samples and the sample of Co_0.9_Yb_0.1_MoO_4_ at 450 °C.

Preliminary investigations of Co_0.9_R_0.1_MoO_4_ (where R = Ho, Yb, Gd) powders suggest that these oxide materials may hold considerable promise for future applications. Their unique properties appear to offer opportunities in areas that have not been extensively explored, potentially contributing to the modernization and advancement of multiple industries.

## Figures and Tables

**Table 1:** Percentage and cell parameters [Å, degree] and volume [Å^3^] of the unit cell for Co_0.9_R_0.1_MoO_4_ (R = Ho, Yb, Gd) phases were observed directly after synthesis (GNP) and in samples that have been calcinated at 450 and 1100 °C, respectively. The data were obtained using the software package Powder Cell 2.4 [36,37] and compared with data from reference [30] marked with *.

Phase	Synthesized	450 °C	1100 °C
**Co_0.9_Ho_0.1_MoO_4_** ICSD card #78328	a = 10.43 b = 9.63 c = 7.06 β = 105.33 V = 683.67 **85.44%**	a = 10.39 b = 9.43 c = 7.06 β = 107.33 V = 660.12 **93.49%**	a = 10.49 b = 9.53 c = 7.16 β = 106.34 V = 686.69 **5.95%**
**Co_0.9_Ho_0.1_MoO_4_** ICSD card #23808	a = 9.77 b = 8.95 c = 7.86 β = 114.82 V = 623.43 **14.56%**	a = 9.65 b = 8.78 c = 7.70 β = 112.83 V = 600.88 **6.51%**	a = 9.61 b = 8.86 c = 7.66 β = 112.82 V = 601.71 **94.05%**
Rp	6.48	7.25	10.30
Rwp	8.23	9.95	14.74
Rexp	0.24	0.5	0.91
**Co_0.9_Yb_0.1_MoO_4_** ICSD card #78328	a = 10.39 b = 9.61 c = 7.06 β = 105.33 V = 679.45 **86.26%**	a = 10.52 b = 9.56 c = 7.07 β = 106.70 V = 680.81 **96.10%**	a = 10.39 b = 9.43 c = 7.10 β = 107.33 V = 663.81 **89.30%**
**Co_0.9_Yb_0.1_MoO_4_** ICSD card #23808	a = 9.77 b = 8.95 c = 7.78 β = 114.82 V = 617.08 **13.74%**	a = 9.63 b = 8.90 c = 7.69 β = 113.88 V = 602.48 **3.90%**	a = 9.57 b = 8.90 c = 7.66 β = 112.82 V = 601.64 **10.70%**
Rp	4.92	8.86	14.08
Rwp	6.30	11.84	22.91
Rexp	0.22	0.35	1.05
**Co_0.9_Gd_0.1_MoO_4_** ICSD card #78328	a = 10.39 b = 9.43 c = 7.07 β = 106.74 V = 663.28 **87.20%**	a = 10.46 b = 9.43 c = 7.06 β = 107.33 V = 664.33 **94.97%**	a = 10.39 b = 9.43 c = 7.08 β = 107.31 V = 662.45 **38.27%**
**Co_0.9_Gd_0.1_MoO_4_** ICSD card #23808	a = 9.76 b = 8.76 c = 7.86 β = 114.82 V = 609.69 **12.80%**	a = 9.76 b = 8.94 c = 7.69 β = 112.82 V = 618.59 **5.03%**	a = 9.71 b = 8.85 c = 7.66 β = 114.08 V = 601.35 **61.73%**
Rp	5.14	9.15	9.33
Rwp	6.69	12.79	13.53
Rexp	0.23	0.38	0.70
CoMoO_4_ * ICSD card #78328	a = 10.57 b = 9.63 c = 7.21 β = 106.59 V = 703.08 **100%**	a = 10.57 b = 9.62 c = 7.22 β = 107.33 V = 700.84 **100%**	/
CoMoO_4_ * ICSD card #23808	/	/	a = 9.71 b = 8.85 c = 7.66 β = 114.10 V = 600.70 **100%**
Rp	3.53	4.21	4.12
Rwp	4.49	5.60	6.05
Rexp	0.17	0.21	0.32

**Table 2:** The results of the quantitative analysis from EDS spectra of Co_0.9_R_0.1_MoO_4_ (R = Ho, Yb, Gd) samples.

**Oxides (wt.%)**		**Oxides (wt.%)**		**Oxides (wt.%)**	
CoO	26.49	CoO	26.54	CoO	26.64
MoO_2_	66.58	MoO_2_	66.48	MoO_2_	66.82
Ho_2_O_3_	6.93	Yb_2_O_3_	6.99	Gd_2_O_3_	6.53
**Elements (at.%)**		**Elements (at.%)**		**Elements (at.%)**	
Co	38.82	Co	38.95	Co	38.91
Mo	57.15	Mo	57.15	Mo	57.15
Ho	4.03	Yb	3.90	Gd	3.94
Ho/(Ho + Co)	9.4	Yb/(Yb + Co)	9.1	Gd/(Gd + Co)	9.2

**Table 3:** Dominant wavelength (Dc) and purity of the color (Pc) of investigated synthesized (synt.), calcinated at 450 °C and 1100 °C samples.

Sample		Dc (nm)	Pc (%)
Co_0.9_Ho_0.1_MoO_4_	synt.	427	99.8
	450 °C	589	4.4
	1100 °C	473	38.6
Co_0.9_Yb_0.1_MoO_4_	synt.	456	65.3
	450 °C	585	78.7
	1100 °C	472	32.8
Co_0.9_Gd_0.1_MoO_4_	synt.	469	13.1
	450 °C	577	7.4
	1100 °C	475	20.3

**Table 4:** The specific surface area (*S*_BET_), mesopore including external surface area (*S*_meso_), specific micropore volume (*V*_mic_), pore volume (*V*_total_), median pore radius (*R*_med_), and maximum pore radius (R_max_) of the Co_0.9_R_0.1_MoO_4_ (R = Ho, Yb, Gd) samples.

Sample	*S*_bet,_ (m^2^/g^−1^)	*S*_meso,_ (m^2^/g^−1^)	*V*_mic_ (cm^3^/g^−1^)	*V*_total_ (cm^3^/g^−1^)	*R*_med,_ (nm)	*R*_max,_ (nm)
Co_0.9_Ho_0.1_MoO_4_ synt.	14.879	8.589	0.0119	0.0433	9.0389	7.1224
Co_0.9_Yb_0.1_MoO_4_ synt.	10.424	11.94	0.0113	0.0077	/	0.3195
Co_0.9_Gd_0.1_MoO_4_ synt.	11.627	15.88	0.0139	0.012	11.737	4.396
Co_0.9_Ho_0.1_MoO_4_ (450 °C)	3.614	19.78	0.0116	0.0072	10	7.4783
Co_0.9_Gd_0.1_MoO_4_ (450 °C)	16.215	1.54	0.0089	0.0443	11.002	8.6617

## Data Availability

The original contributions presented in this study are included in the article. Further inquiries can be directed to the corresponding author.
